# Gut microbiota translocation contributes to early islet apoptosis in streptozotocin-induced diabetes

**DOI:** 10.1128/msystems.00172-26

**Published:** 2026-06-22

**Authors:** Ying Wang, Peng Sheng, Shijia Wang, Xiaohui Zhong, Hong Cao, Dan Li, Jiai Yan, Ju Yang, Yingyu Wang, Jie Peng, Fengping Sun, Shunhe Wang, Yongwei Feng, Jing Sun, Feng Zhang

**Affiliations:** 1 Department of Nutrition, Affiliated Hospital of Jiangnan University 199193, Wuxi, China; 2 Wuxi School of Medicine, Jiangnan University 66374, Wuxi, China; 3 Institute of Future Food Technology, JITRI 650668, Yixing, China; 4 Department of Clinical Nutrition, The Second Affiliated Hospital of Soochow University 105860, Suzhou, China; 5 College of Health and Nursing, Wuxi Taihu University 164393, Wuxi, China; 6 School of Bioengineering, Jiangnan University 66374, Wuxi, China; 7 Department of Endocrinology, Affiliated Hospital of Jiangnan University 199193, Wuxi, China; 8 School of Food Science and Technology, Jiangnan University 66374, Wuxi, China; 9 Wuxi Food Safety Inspection and Test Center, Technology Innovation Center of Special Food for State Market Regulation, Wuxi, China; 10 School of Environment and Ecology, Jiangnan University 66374, Wuxi, China; University of South Florida Morsani College of Medicine, Tampa, Florida, USA; University of Pennsylvania, Philadelphia, Pennsylvania, USA

**Keywords:** gut microbiota, pancreatic islet apoptosis, intestinal barrier, diabetes

## Abstract

Dysbiosis of the gut microbiota and impaired intestinal barrier are associated with diabetes development. The translocation of gut microbiota induced by streptozotocin (STZ) has been confirmed to damage pancreatic islets. However, it remains uncertain whether dysregulated gut microbiota plays an essential role in the translocation leading to pancreatic injury. In specific pathogen-free (SPF) and germ-free (GF) mice treated with STZ, we measured glucose metabolism levels, pancreatic islet damage, intestinal barrier integrity, and bacterial content in the pancreas to investigate the role of gut microbiota translocation in diabetes development. Shotgun metagenomic sequencing was used to analyze the impact of STZ on gut microbiota structure and function. Fecal microbiota transplantation was performed to explore if gut microbiota translocation depends on STZ-induced structural dysregulation. STZ induced intestinal damage in SPF mice, resulting in gut microbiota translocation to the pancreas, pancreatic apoptosis, and dysregulated glucose metabolism. Despite inherent intestinal barrier damage, absence of pancreatic apoptosis in GF mice further indicates that gut microbiota translocation is an essential prerequisite for STZ-induced pancreatic islet apoptosis. STZ significantly altered mouse gut microbiota composition and function. Transplantation of fecal microbiota from STZ-treated or saline-treated mice into STZ-induced GF mice also resulted in microbial translocation and pancreas apoptosis. Apoptosis of β cells in STZ-treated mice results from gut microbiota translocating to the pancreas through impaired intestinal barrier caused by STZ treatment independent of alterations in the gut microbial community.

**IMPORTANCE:**

In our study, the apoptosis of β cells in STZ-treated mice is the result of the translocation of gut microbiota to the pancreas through the impaired intestinal barrier induced by STZ, independent of alterations in the gut microbiota. These findings proposed the potential role of compounds in impairing the intestinal barrier integrity, promoting microbiota migration and finally damaging pancreatic islets.

## INTRODUCTION

Diabetes mellitus is one of the most common metabolic diseases caused by insufficient insulin secretion and/or insulin resistance (1). Type 2 diabetes mellitus (T2DM) is a complex, chronic metabolic disorder characterized primarily by insulin resistance and gradual β-cell dysfunction. Loss of pancreatic β cells is a key driver in the onset of diabetes mellitus, a process often accompanied by endoplasmic reticulum stress within these cells, which ultimately contributes to disease progression (2–5). Despite the influence of genetic factors, environmental elements, such as endocrine-disrupting chemicals, viral infections, and unhealthy diets, also play a significant role in pancreatic β-cell damage and contribute to the development of diabetes (6–8). Besides damaging pancreatic β cells (9), environmental endocrine-disrupting chemicals, such as polychlorinated biphenyls, arsenic and cadmium, have also been discovered to impair intestinal permeability (10).

The gut microbiota has been considered as an important influencing factor involving in the development or alleviation of metabolic diseases, such as diabetes, obesity, and metabolic dysfunction-associated steatotic liver disease (11). The detrimental gut bacterial metabolites, such as trimethylamine (12), lipopolysaccharide (13), and aromatic amino acids (14), drive the development of diabetes by disrupting key metabolic pathways. Recent studies have shown that in dextrose sodium sulfate treated mice, gut bacteria could migrate to the pancreas, resulting in local inflammation and destruction of β cells (15). In addition, diabetic patients often exhibit impaired intestinal barrier function (16) and the gut microbiota dysbiosis (17). An intact intestinal barrier serves as a vital defense, protecting the host from the detrimental effects of gut microbiota, food antigens, and gut-derived toxins (18). Therefore, the translocation of the gut microbiota may potentially impair the pancreatic β cells in patients with diabetes.

STZ-induced mouse model is commonly used to study the progression of diabetes. STZ further damages the DNA of the pancreatic cells by inducing pancreatic inflammation (19), leading to the apoptosis of pancreatic β cells (20). This process is similar to the inflammation in the pancreas of diabetic patients. In a recent study, STZ was found to be able to induce the translocation of gut microbiota to the pancreatic islet, leading to the damage of β cells (21). However, whether the dysregulated gut microbiota is an essential prerequisite for STZ-induced pancreatic apoptosis remains uncertain.

The aim of this study is to investigate whether STZ-induced changes in the gut microbiota is the critical factor leading to pancreatic islet apoptosis through microbiota translocation. Surprisingly, we found that STZ-induced apoptosis of pancreatic β-cells in diabetic mice was caused by the translocation of gut microbiota to the pancreas through the compromised intestinal barrier, independent of changes in gut microbiota.

## MATERIALS AND METHODS

### Animals

Four-week-old, male-specific pathogen-free (SPF) and germ-free (GF) C57BL/6J mice were obtained from the Gempharmatech Co., Ltd (Nanjing, Jiangsu, China).

Conventional housing conditions were SPF conditions, under which C57BL/6J mice were housed in a SPF barrier facility with a controlled environment accredited. The GF mice used in the experiment were maintained in sterile cages, the sterile cages were placed in a large isolator, and the drinking water was sterilized under high pressure, placed in the aseptic tube, and butted with the isolator. All mice were co-housed with two animals per cage to minimize the number of variables and to standardize the early-life microbial exposure within each experimental unit. The feed was irradiated, placed in the delivery bin, sterilized by chlorine dioxide spray for 12 h, and transferred into the isolator. Surveillance for bacterial contamination was performed by periodic bacteriological examinations of feces. The mice were fed with high-fat diet (60% energy from fat; D12492, Xietong Pharmaceutical Bio-engineering Co., Ltd.) and were housed in a room maintained at 22.0°C ± 1°C with 30%−70% humidity, with a 12-h light/dark phase cycle (lights on at 07:00 am). Surveillance for bacterial contamination was performed by periodic bacteriological examinations of feces, food, and padding.

### Study design

#### Trial-1

**Fig 1:**
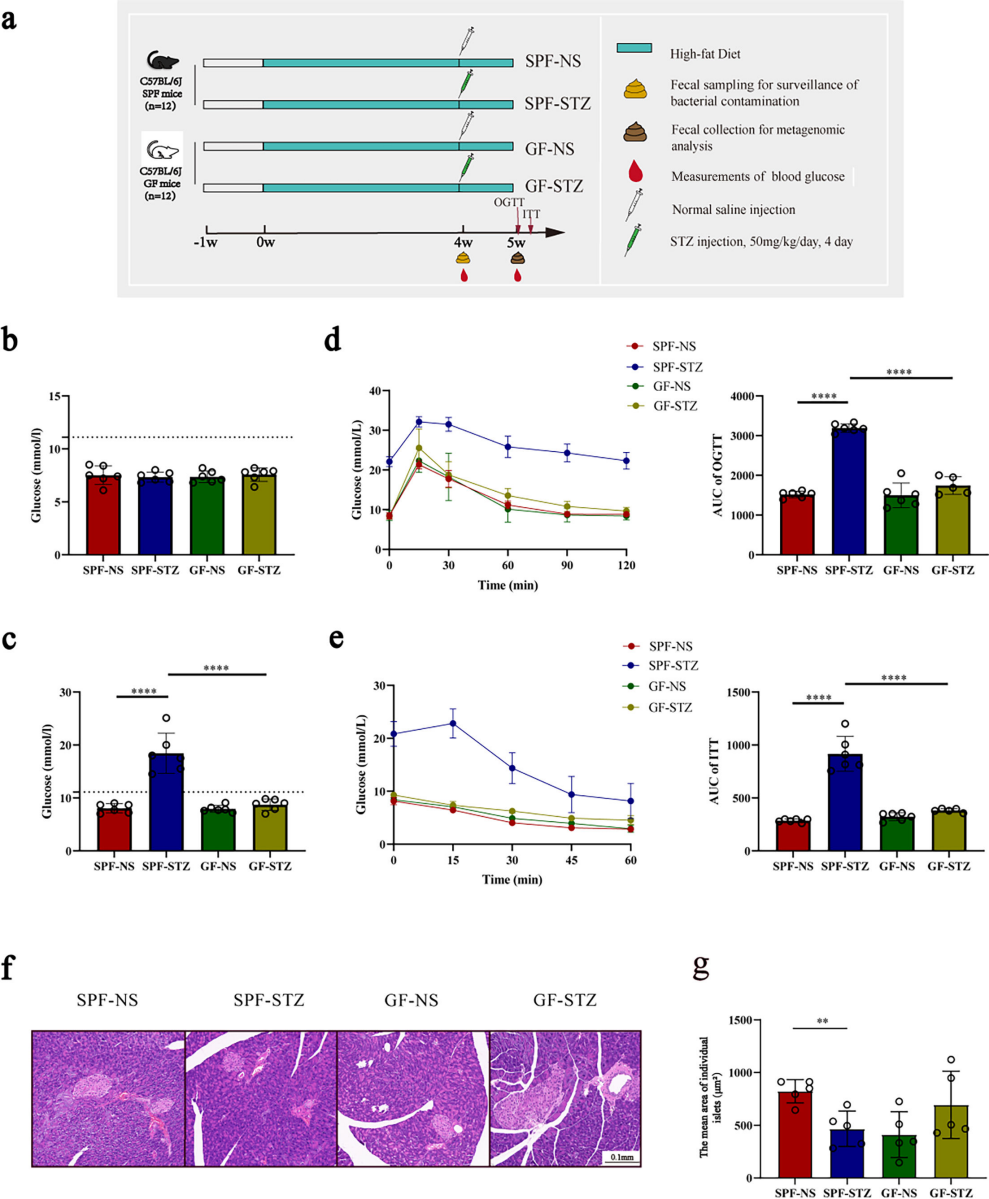
Dysbiosis of glucose metabolism and pancreatic tissue injury after STZ injection in mice. (**a**) Experimental design. (**b**) FBG level of mice after 4 weeks of high-fat diet. (**c**) FBG level of mice on the fourth day after the accompolishment of STZ injection. (**d**) OGTT and its AUC before the sacrifice. (**e**) ITT and its AUC before the sacrifice. (**f**) H&E staining in pancreatic tissue. Scale, 0.1 mm. (**g**) The mean area of individual islets. Data were normalized to control expression level. The data were presented as means ± SEM. The significance of the differences was determined by one-way ANOVA. Bars that did not share the same letters were significantly different (*P < 0.05*) from each other, *n* = 5.

After 4-week high-fat diet, SPF mice (*n* = 12) and GF mice (*n* = 12) were divided into four groups (SPF-NS, SPF-STZ, GF-NS, and GF-STZ), which received intraperitoneal inoculation of STZ (18883-66-4, Sigma-Aldrich, USA) at a dose of 50 mg/kg (22) or normal saline (NS), respectively, for four consecutive days. The blood glucose level was measured on the fourth day after the completion of STZ injection. Stool samples collected at week 4 were used to monitor whether the feces of GF mice were contaminated by bacteria. Stool samples collected at week 5 were used for metagenomic analysis. Subsequently, oral glucose tolerance tests (OGTTs) and insulin tolerance test (ITT) were conducted before mice were dissected, and their tissues were preserved. The experimental grouping is illustrated in Fig. 1a.

#### Trial-2

Following a 4-week high-fat diet, mice were assigned to six experimental groups: SPF-NS_NS_: SPF mice receiving i.p. normal saline, followed 1 week later by oral gavage of saline; SPF-STZ_NS_: SPF mice receiving i.p. STZ, followed 1 week later by oral gavage of saline; GF-NS_NS_: GF mice receiving i.p. normal saline, followed 1 week later by oral gavage of saline; GF-STZ_NS_: GF mice receiving i.p. STZ, followed 1 week later by oral gavage of saline; GF-STZ _FMT-NS_: GF mice receiving i.p. STZ, followed 1 week later by oral gavage of fecal microbiota from fresh feces from the SPF-NS_NS_ group; GF-STZ _FMT-STZ_: GF mice receiving i.p. STZ, followed 1 week later by oral gavage of fecal microbiota from fresh feces from the SPF-STZ_NS_ group. Blood glucose levels were measured at three time points: on day 4 after the final i.p. injection, and at 1 and 2 weeks after FMT. Stool samples collected at week 4 were used to monitor whether the feces of GF mice were contaminated by bacteria, and stool samples collected at week 7 were employed for quantitative analysis of monosaccharides and oligosaccharides. Subsequently, OGTT and ITT were conducted before mice were dissected, and their tissues were preserved. The experimental grouping is illustrated in Fig. 4a. Notably, STZ administration led to mortality in 3/7 (GF-STZ_NS_) and 1/7 (GF-STZ _FMT-STZ_) mice. Necropsy identified mild lung swelling, intestinal autolysis, and hardened adipose tissue, potentially due to the lack of gut microbiota-derived protection.

### Plasma measurements

Once a week, after fasting for 8 h, glucose levels in the blood of the mice tail vein were measured with a glucose meter (Roche Diagnostics GmbH, Mannheim, Germany). Centrifugation tubes containing blood samples were centrifuged for 20 min at 1,000 × *g* at 4°C. Plasma was aliquoted and stored at −80°C until further measurements. The serum insulin levels were determined by ELISA kits (E-EL-M2614c, Elabscience, China). The levels of total protein (TP), albumin (ALB), globulin (GLOB), total cholesterol (TC), and triglycerides (TG) were determined using an automated blood lipid analyzer (Beckman Pipeline Biochemistry Instrument: AU5800) in serum.

### HOMA-β

HOMA-β is an index used to evaluate the function of pancreatic β cells in individuals. It is calculated as follows:

20 × fasting insulin (μU/mL) / [fasting blood glucose (mmol/L) −3.5]% (23).

### Oral glucose tolerance tests (OGTTs) and insulin tolerance test (ITT)

The OGTT and ITT were performed according to a previously reported method (24). For OGTT, mice were subjected to a 16-h fasting period during their nocturnal phase and subsequently administered with a gavage of 20% glucose (1 g/kg body weight). Blood glucose levels were then assessed at 0, 15, 30, 60, 90, and 120 min post-administration. In the case of the ITT, mice underwent a morning fast lasting for 4 h before receiving intraperitoneal injection of human insulin (Humalog, 0.75 units/kg). Blood glucose levels were measured at intervals of 0, 15, 30, 45, and 60 min following administration.

### Histological analysis

Intestines and pancreas were collected and then fixed for 24–36 h in 10% (v/v) formalin and subsequently embedded in paraffin. Paraffin-embedded tissues were micro-sectioned at a thickness of 4 μm. The slides were stained with H&E using a standard procedure. The images were acquired using a Zeiss AxioObserver Z1 microscope. The acquired images were then quantified using ImageJ software.

### Histological measurement of intestinal injury

The segment of small intestine was stained with hematoxylin-eosin. Histologic changes were assessed using light microscopy by two independent observers, who were blinded to the study design, and the degree of intestinal mucosal injury was assessed quantitatively using Chiu’s score on a scale from 0 (normal) to 5 (severe injury) (25).

### Shotgun metagenomics sequencing

Total genomic DNA from fecal samples was extracted using the E.Z.N.A. Soil DNA Kit (Omega Bio-tek, Norcross, GA, USA) according to the manufacturer’s instructions. After extraction, the concentration and purity of the extracted DNA were determined with TBS-380 and NanoDrop 2000, respectively, and quality was checked on 1% agarose gel. DNA was fragmented to an average size of about 300 base pairs (bp) using Covaris M220 for paired-end library construction. The paired-end library was constructed using NEXTFLEX Rapid DNA-Seq (BioScientific, Austin, TX, USA). Paired-end sequencing was performed on Illumina HiSeq (Illumina Inc., San Diego, CA, USA) using standard protocols in Shanghai Hongxu Bio-pharm Technology Co. Ltd.

Raw fastq files were quality filtered using Sickle, and low-quality reads (length <50 bp or with a quality value <20 or having N bases) were removed. Reads were aligned to the human genome by the Burrows-Wheeler Aligner, and any hit associated with the reads and their mated reads were removed. Metagenomics data were assembled using MEGAHIT, and contigs with a length of 300 bp or more were selected as the final assembly result. Open reading frames from each assembled contig were predicted using Metagene (26). All predicted genes with a 95% sequence identity were clustered using CD-HIT. Reads after quality control were mapped to the representative sequences with 95% identity using SOAPaligner.

The KEGG annotation was conducted using BLASTP against the database with an e-value cutoff of 1 × 10^−5^. PCoA was used to visually evaluate the overall difference and similarity of microbiota communities between the SPF-NS and SPF-STZ groups. The PERMANOVA was used to test group differences. The differential bacterial species between the two groups were identified using LEfSe, employing a linear discriminant analysis (LDA). The reads used for further analysis are shown in Tables S1 to S4.

### Bacterial load in the pancreas

Pancreas collection for SPF and GF mice was performed using aseptic instruments and containers under clean conditions. Operators wore masks and sterile gloves. After the pancreatic tissue had been collected, it was placed in liquid nitrogen immediately and then preserved in −80°C for the further detection. Pre-cut pancreatic tissue (20 mg) was homogenized using a sterile pestle in a sterile mortar under liquid nitrogen as the media. Simultaneously with the excision of the pancreas, an empty EP tube was opened as a negative control, and subsequent procedures perform on the control were conducted in an identical manner to procedures performed on the pancreas. Homogenate was used for DNA extraction by QIAamp Pro DNA Kit (51804, Qiagen, Duesseldorf, Germany). The experiment was conducted on ice, with DNA extraction and PCR amplification operations performed in separate laboratory areas to minimize the risk of cross-contamination.

The PCR was performed as previously described (15). The 16S rRNA gene was frst amplifed using the primers 27-F (AGAGTTTGATCCTGGCTCAG) and 1492-R (GGTTACCTTGTTACGACTT). PCR was performed in a volume of 25 μL with 10 ng DNA template, 1 μL of each primer (12.5 μM), 2.5 μL of 10× PCR buffer, 0.3 μL of Taq polymerase (TaKaRa), 1 μL of dNTP mix (2.5 mM). The reaction conditions consisted of initial denaturation at 94°C for 5 min; 40 cycles of 94°C for 30 s, 55°C for 30 s and 72°C for 90 s; and a final extension step for 10 min at 72°C. Subsequently, a 46- bp length sequence located in 16S rRNA gene was used for quantitative PCR through the primers Uni331F (TCCTACGGGAGGCAGCAGT) and Uni797R (GGACTACCAGGGTATCTAATCCTGTT) (27). Briefly, qPCR was performed with the Light Cycler 96 (Roche, Geneva, Switzerland) using iQ SYBR Green Supermix (170-8882AP, BIO-RAD, CA, USA). The PCR conditions were 95°C for 3 min, followed by 40 cycles of 95°C for 15 s, 60°C for 60 s, and plate read for 5 s at 80°C.

### FMT preparation

For the FMT protocol, all fecal samples were processed under anaerobic conditions. The FMT was performed, as previously reported (28). Fecal slurries were prepared by collecting fresh fecal matter pellets (5 mg) from the SPF-STZ_NS_ and SPF-NS_NS_ mice respectively and mixing with 1 mL of sterile water. The feces were homogenized in the water using a dounce homogenizer. Then pooled samples were centrifuged at 100 × *g* for 2 min. After allowing the suspension to settle for 5 min the supernatant was collected. STZ-induced GF mice were colonized with fecal bacteria from either SPF-NS_NS_ or SPF-STZ_NS_ mice by oral gavage (200 µL).

### Western blot analysis

Proteins were collected, and Western blot was used to analyze protein expression, as previously reported (29). Total protein was lysed by RIPA Lysis Buffer (P0013B, Beyotime, Shanghai, China) containing 1% PMSF (ST507, Beyotime, Shanghai, China) and 1% Phosphatase Inhibitor Cocktail I (HY-K0021, MedChemExpress, Shanghai, China). Then, the lysate was centrifuged at 12,000 × *g*, 4°C for 20 min to obtain the total protein. The protein concentrations were detected by the BCA Protein Assay Kit (P1511, APPLYGEN, China). Protein content was separated by 12% sodium dodecyl sulfate-polyacrylamide gel electrophoresis (SDS-PAGE) (G2003-50T, Servicebio, Wuhan, China) and then transferred onto polyvinylidene fluoride (PVDF) (HATF09025, Millipore, Massachusetts, USA) membranes. These membranes were then blocked with 5% non-fat dry milk in TBST (Tris-buffered saline with 0.05% Tween-20) for 2 h. Subsequently, membranes were probed with primary antibodies: GLUT2 (1:1,000, A12370, Abclonal, Wuhan, China), Claudin-1 (1:1,000, Ab211737, Abcam, Cambridge UK), β-actin (1:5,000, AF7018, Affinity, USA), Bax (1:2,000, A19684, Abclonal, Wuhan, China) and Bcl-2 (1:1,000, 15071S, Cell Signaling Technology, USA) overnight at 4°C. After that, the membranes were incubated at room temperature for 2 h with diluted secondary antibodies against rabbit (1:5,000, A0208, Beyotime, Shanghai, China) or mouse (1:3,000, A0216, Beyotime, Shanghai, China) primary antibodies. Blots were detected by ECL + Plus reagents (Yeasen, Shanghai, China) and imaged by Tanon-5200Multib instrument (Shanghai, China). The density of target protein was quantified by ImageJ Software.

### Metabolome analysis

GC-MS was performed as previously reported with minor modifications (30). Before conducting qualitative and quantitative analyses of monosaccharides and oligosaccharides in mice stool, about 15 mg of mouse feces powder was weighed. The material was added to a 2-mL centrifugation tube, and then 1 mL of 80% methanol/water under ultrasonic waves at room temperature for 1 h. The extraction was carried out overnight at 4°C. On the second day, the residue was once again extracted with 1 mL of 80% methanol/water, and the two extracts were combined as the crude extract of free monosaccharides and oligosaccharides. Subsequently, sugar derivatization was conducted by adding 20 mg/mL methoxyamine hydrochloride/pyridine to a 50-µL sample at 40°C for 2 h, followed by the addition of BSTFA or MSTFA (both derivatives) to an 80-µL aliquot at 40°C for 1 h. Next, 100-µL hexane was introduced before injecting a final volume of 1 µL. Finally, soluble monosaccharides and disaccharides were subjected to qualitative and quantitative analysis using GC-MS (7890A, Agilent, USA). The gas chromatography column used for this study was an HP-5ms (30 m, 0.25-mm internal diameter, 0.25-mm film coating; P.J. Cobert). A linear temperature gradient was used. The initial temperature of 80°C was held for 5 min and increased to 210°C over a period of 22 min. Then, the temperature was raised from 210°C to 300°C in 1 min. The temperature was held at 300°C for 5 min. The samples were run by electron ionization, and the source temperature, electron energy, and emission current were 200°C, 70 eV, and 300 mA, respectively. The injector and transfer line temperatures were 250°C.

### Statistical analyses

Results were expressed as mean ± SEM. Statistical analysis was conducted using GraphPad Prism software (GraphPad Software, San Diego, CA). Data without indications were analyzed by Student’s *t*-test. Statistical significance is reported as *, *P* ≤ 0.05, **, *P* ≤ 0.01, ***, *P* ≤ 0.001, and ****, or *P* ≤ 0.0001. The metagenomic data of gut microbiota were analyzed and mapped using R language (version 3.2.2).

## RESULTS

### STZ-treated GF mice did not exhibit hyperglycemia or islet damage

SPF and GF C57BL/6J mice were employed to investigate the role of the gut microbiota in STZ-induced hyperglycemia. After a 4-week high-fat diet, SPF and GF mice were assigned into four groups (SPF-NS, SPF-STZ, GF-NS, and GF-STZ groups), which were inoculated intraperitoneally with multiple low doses of STZ or NS, respectively, for four consecutive days (Fig. 1a). The glucose metabolism level and the number and area of the pancreatic islets were assessed on the fourth day after the accompolishment of STZ injection. After a high-fat diet for 4 weeks, there was no difference in fasting blood glucose (FBG) levels among the four groups of mice (Fig. 1b). On the fourth day after the accompolishment of STZ injection, FBG levels in SPF mice were significantly elevated compared with mice injected with NS (Fig. 1c), with all values meeting the criteria for diabetes (FBG ≥ 11.1 mmol/L). Conversely, there was no significant difference in FBG levels between GF mice injected with STZ and NS. Before the sacrifice, the values of area under the curve (AUC) of OGTT and ITT were significantly higher in the SPF-STZ group compared with the SPF-NS group, whereas no significant difference was observed between the GF-STZ group and GF-NS group (Fig. 1d and e). These findings suggest that STZ-induced glucose metabolism disorders depend on the participation of gut microbiota.

The hyperglycemia induced by STZ is attributed to DNA damage in the pancreatic islet, subsequently leading to pancreatic apoptosis and consequent elevation of blood glucose levels (20). Histological examination of the pancreas showed that in the SPF-NS group, pancreatic tissues exhibited normal appearance with well-organized islet cells and clear demarcation between the islets and surrounding tissues. In contrast, the pancreas of mice in the SPF-STZ group displayed evident histopathological damage characterized by blurred boundaries and even atrophy of islets. The pancreatic structures in the GF-NS and GF-STZ mice showed comparable clarity and intact islets (Fig. 1f). The number and relative area of islets were significantly lower in the SPF-STZ group compared with the SPF-NS group (Fig. 1g and h). However, no significant differences could be observed in terms of the number and relative area of islets between the GF-NS and GF-STZ group. These results demonstrate that the presence of gut microbiota play an important role in the process of STZ-induced pancreatic islet injury in mice.

### STZ-induced pancreatic islet injury was related with translocation of the gut microbiota

**Fig 2:**
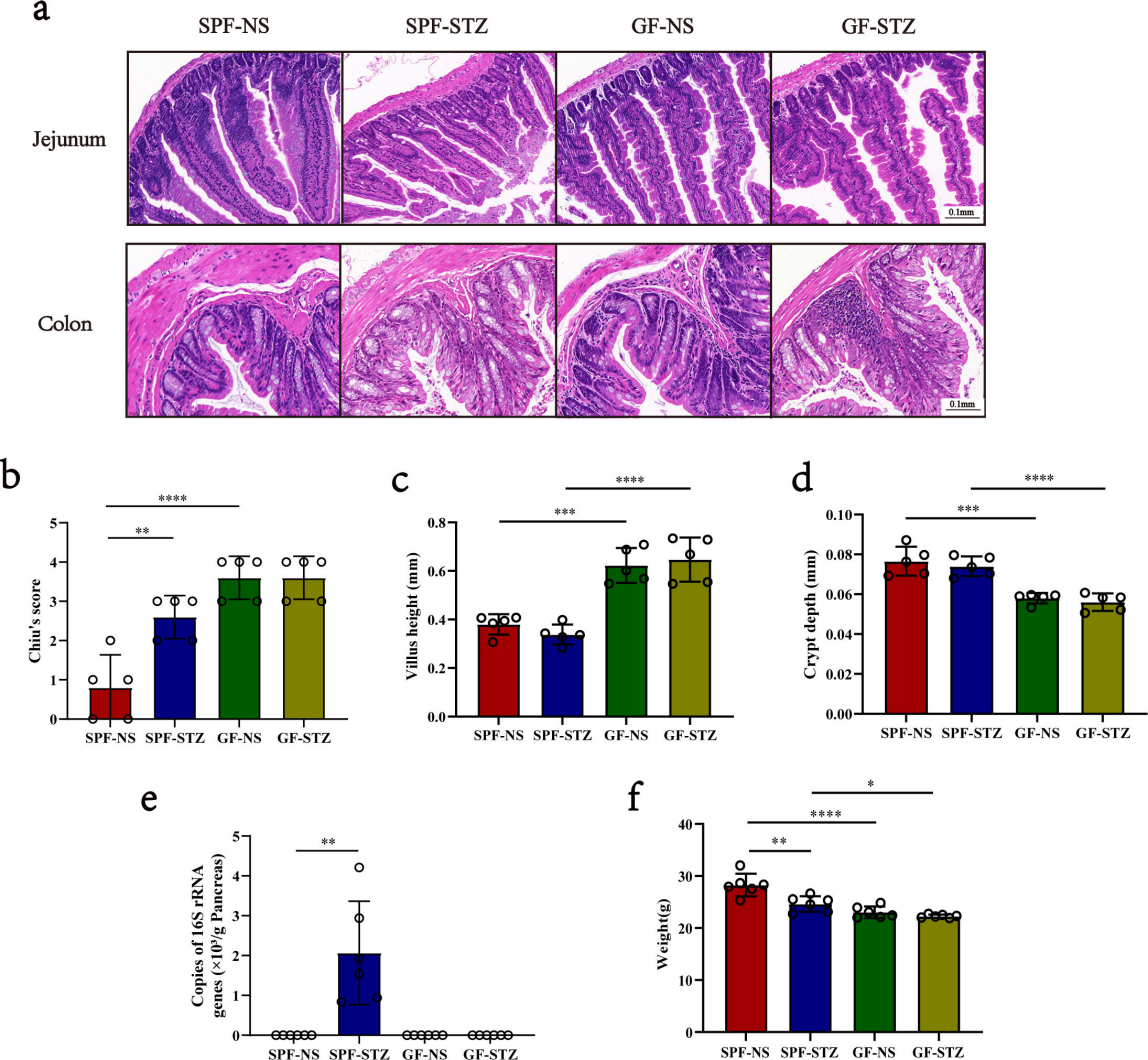
STZ-induced pancreatic islet injury was related with translocation of the gut microbiota. (**a**) H&E staining of jejunum and colon. Scale, 0.1 mm. (**b**) Chiu’s score of the jejunum. (**c**) The height of the villi in the jejunum. (**d**) The depth of the crypt in the jejunum. (**e**) The bacterial content in the pancreas was analyzed by qPCR. (**f**) Body weight of mice after injection of STZ, *n* = 5.

After 1 week of STZ injection in SPF mice, there was a significant alteration in the histological morphology of the intestine (Fig. 2a). The small intestine showed significant damage and villous atrophy in SPF-STZ mice. There was no discernible distinction in the appearance of the small intestine between GF-STZ and GF-NS groups. The small intestinal villi of GF mice (GF-NS and GF-STZ) showed elongation (Fig. 2c), while concurrently displaying severe epithelial damage, atrophic villi, pronounced barrier impairment, crypt deformation and significantly reduced crypt height (Fig. 2d). The result of small intestine Chiu’s injury score indicated that the SPF-STZ group exhibited a higher score compared with the SPF-NS group, thereby demonstrating the ability of STZ to induce injury in the small intestinal epithelium. No significant difference was observed between the GF-STZ and GF-NS groups. However, Chiu’s injury scores of GF mice were generally elevated in comparison to those of SPF mice (Fig. 2b). The colonic histology analysis revealed disrupted colonic structure and infiltration of inflammatory cells in the mucous layer and lamina propria of SPF-STZ mice. The colonic structure displayed no discernible difference between the GF-STZ and GF-NS groups. However, GF mice was characterized by the impaired colonic architecture, reduced goblet cell population, which was consistent with findings observed in the small intestine. These results revealed that STZ-induced islet injury in mice was accompanied by intestinal injury.

To evaluate whether gut microbiota translocation contributes to STZ-induced injury of pancreatic islet, qPCR was used to determine the bacterial content in pancreas. Significant increase in bacterial content was detected within the pancreas of the SPF-STZ group compared with the SPF-NS group. This finding combined with the impaired intestine suggested an involvement of bacterial migration in STZ-induced islet injury (Fig. 2e), which was consistent with other studies (21). Absence of the bacterial migration and impairment in pancreas in GF mice, even when the intestine was injuried, suggested that the damage to pancreatic islets induced by STZ may depend on the translocation of the gut microbiota.

Considering the extensive damage observed in the small intestine of GF mice, it is postulated that nutrient malabsorption occurred, which exerted additional influence on glucose metabolism (31). The SPF-STZ group exhibited significantly lower body weight compared with the SPF-NS group, while no significant difference was observed between the GF-STZ and GF-NS groups. Furthermore, GF mice all displayed lower body weight than SPF mice (Fig. 2f). The concentrations of total protein, albumin, globulin, cholesterol and triglyceride in serum were detected. No statistically significant disparity could be found in these serum indexes between SPF-STZ and SPF-NS mice. However, the levels of total protein, albumin, globulin, and cholesterol were significantly lower in GF mice compared with SPF mice (Fig. S1a through e). These findings suggested that STZ injection had not impact nutrient absorption despite inducing intestinal injury in SPF mice. In contrast, GF mice exhibited significant nutrient malabsorption and intestinal damage whether with STZ injection or not, which may contribute to their unchanged blood glucose following STZ treatment.

### STZ induced the alteration of the gut microbiota in the composition and function

**Fig 3:**
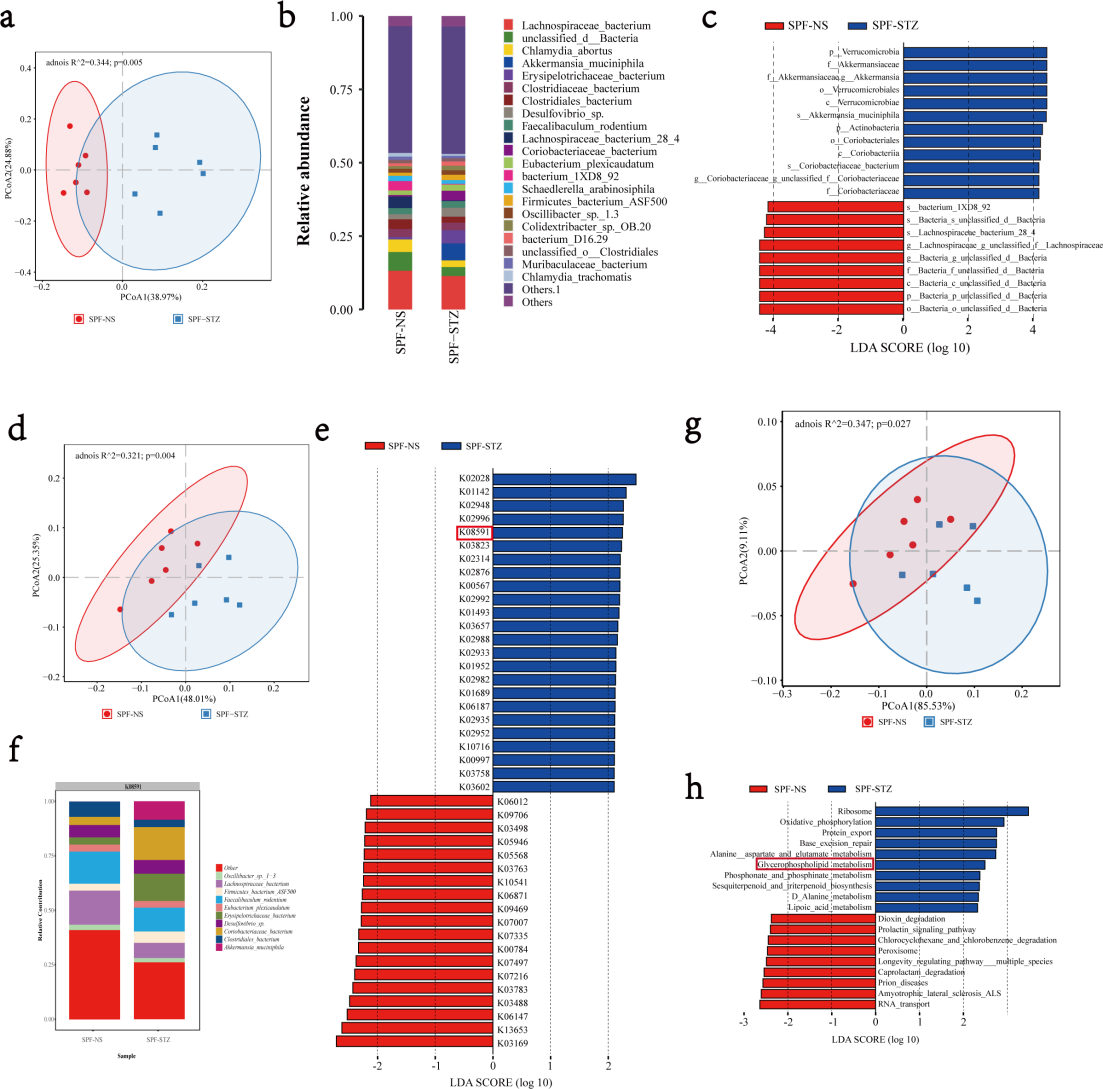
The alteration of the composition and function in the gut microbiota in T2D model. (**a**) Principal component ordination analysis (PCoA) of gut microbiota between the SPF-STZ and SPF-NS groups. (*P = 0.005*). (**b**) Taxonomic composition distribution of gut microbiota at species level in the SPF-STZ and SPF-NS groups. (**c**) Differential enrichment groups identified by linear discriminant analysis effect size (LEfSe) between the SPF-STZ and SPF-NS groups. The length of the columns represents the effect of significantly different species on relative abundance (LDA score [log10] > 4). Red and blue indicate significantly increased bacterial taxa in the SPF-NS and SPF-STZ groups, respectively. (**d**) PCoA of KOs across two groups. (**f**) Bar graph of LDA score of enriched KOs at level 2.2 LDA scores (log 10) >2.2 and *P < 0.05* are shown. (**e**) The proportion of bacterium carrying K08591. (**g**) PCoA of pathway across two groups. (**h**) Bar graph of LDA score of enriched KEGG pathways at LDA scores (log 10) > 2 and *P < 0.05* are shown. Data are provided at mean ± SEM, *n* = 6.

Given the fact that gut microbiota dysbiosis is associated with bacterial translocation in diabetes development, both in humans and mice (32–35). No significant difference was observed in the gut microbiota α-diversity (Shannon index) between the two groups(Fig. S1f). We next assessed the variation of gut microbiota in STZ-induced diabetic mice. Based on the principal component analysis (PCoA), significant disparity was discovered in the taxon, KEGG Orthology (KO) and pathway of gut microbiota between the SPF-STZ and SPF-NS groups (Fig. 3a, e and f). There were obvious differences in the relative abundances of *Lachnospiraceae bacterium 28-4*, *Bacterium 1XD8-92*, *Akkermansia muciniphila*, and Coriobacteriaceae bacterium between the SPF-STZ and SPF-NS groups based on the LEfSe analysis. In the SPF-STZ group, the relative abundances of *A. muciniphila* and Coriobacteriaceae bacterium were higher, while the relative abundances of *Lachnospiraceae bacterium 28-4* and *Bacterium 1XD8-92* were lower compared with those in the SPF-NS group (Fig. 3b and c). Furthermore, the SPF-STZ group revealed the most significant fold increase in the relative abundance of *A. muciniphila* (Fig. 3d).

Subsequently, a comparative analysis was performed between KOs obtained from Kyoto Encyclopedia of Genes and Genomes (KEGG). Based on LEfSe analysis, we focused on those KOs that were closely related with metabolic processes. Significant enrichment in the SPF-STZ group compared with the SPF-NS group was observed for K08591, which encodes glycerol-3-phosphate acyltransferase and yields lysophosphatidic acid as its downstream product (Fig. 3f). However, other KOs promoted or reduced in the SPF-STZ group were found to be unrelated to metabolic processes. Compared with the SPF-NS group, *A. muciniphila* and Coriobacteriaceae bacterium exhibited the most significant increase in abundance among bacteria carrying K08591 in the SPF-STZ group (Fig. 3g).

The gut microbiota in the SPF-STZ group had significant alterations in “ribosome,” “oxidative phosphorylation,” “glutamate and glutamate metabolism,” “glycerophospholipid metabolism,” “phosphate and phosphate metabolism,” as well as “D. Alanine metabolism” and other pathways (Fig. 3i). Notably, the “glycerophospholipid metabolism” pathway contains K08591, both of which increased in the SPF-STZ group.

### STZ induced disorder of glucose metabolism and islet function depends on the presence of gut microbiota

**Fig 4:**
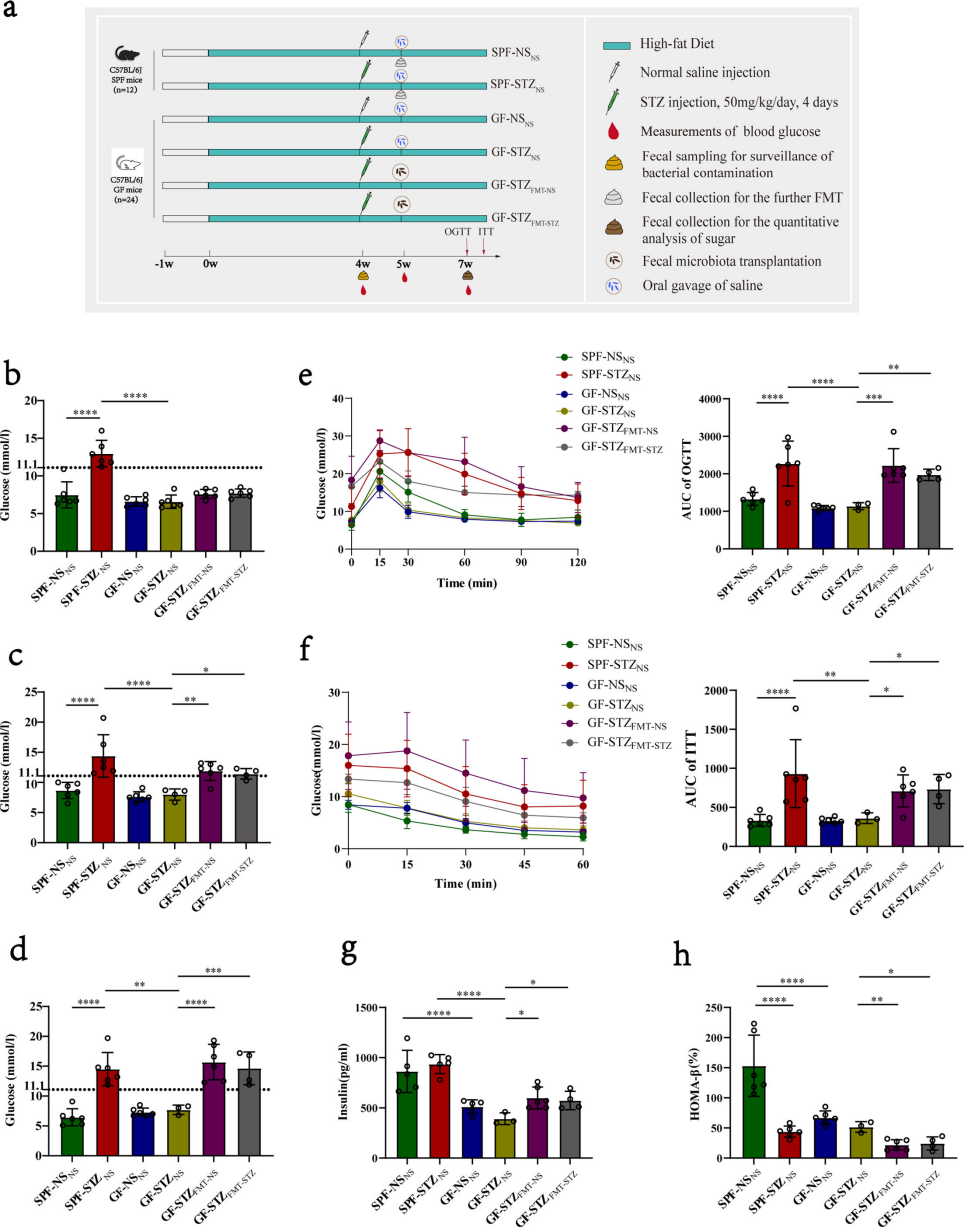
STZ induced disorder of glucose metabolism and islet function in the presence of gut microbiota. (**a**) Experimental design of FMT. (**b**) FBG level of mice on the fourth day after the accomplishment of STZ injection. (**c**) FBG level in each group after 1 week of FMT. (**d**) FBG level in each group after 2 weeks of FMT. (**e**) OGTT and AUC before the sacrifice. (**f**) ITT and AUC before the sacrifice. (**g**) Serum insulin levels measured after dissection at 7 weeks. (**h**) Islet β-cell function index HOMA-β, *n* ≥ 3.

To investigate whether STZ-induced changes of the gut microbiota could aggravate islet damage, fecal microbiota from SPF-STZ and SPF-NS groups in trial one were transplanted respectively into STZ-induced GF mice on the fourth day after the accompolishment of STZ injection (GF-STZ_FMT-STZ_ and GF-STZ_FMT-NS_) (Fig. 4a). Following 4 weeks of high-fat diet, SPF or GF mice were administered STZ or NS (SPF-STZ_NS_, SPF-NS_NS_, GF-STZ_NS_, and GF-NS_NS_). FBG levels showed a significant increase after transplantation into STZ-treated GF mice with fecal microbiota from the SPF-STZ (GF-STZ_FMT-STZ_) and SPF-NS group (GF-NS_FMT-NS_) (Fig. 4c and d). Before the sacrifice, the area under the curve (AUC) of OGTT and ITT in GF-STZ_FMT-STZ_ and GF-STZ_FMT-NS_ groups exhibited significantly higher than GF-STZ_NS_ group, with no difference between two transplantation groups (Fig. 4e and f). These results indicate that the presence of gut microbiota is an essential prerequisite for STZ-induced metabolic disorders, and that STZ-altered microbiota is a consequence rather than a cause of glucose intolerance.

Despite the elevation in FBG levels observed in SPF-STZ_NS_ mice, there was no concomitant increase in serum insulin secretion. Surprisingly, GF mice showed lower insulin secretion compared with SPF mice and mice transplanted with microbiota from the SPF-STZ_NS_ and SPF-NS_NS_ groups (Fig. 4g). As expected, HOMA-β reflecting islet β-cell function was significantly lower in the SPF-STZ_NS_ group compared with the SPF-NS_NS_ group (Fig. 4h). No significant difference in HOMA-β was observed between the GF-STZ_NS_ and GF-NS_NS_ groups. Both the GF-STZ_FMT-STZ_ and GF-STZ_FMT-NS_ groups exhibited significantly lower levels of HOMA-β than the GF-STZ_NS_ group, while no difference was found between these two FMT groups. After fecal microbiota transplantation, the mice exhibited a reduction in HOMA-β compared with the GF-STZ group, indicating that impairment of β-cell function after STZ injection in mice must be assisted by the gut microbiota. HOMA-β of the GF-STZ_FMT-STZ_ and GF-STZ_FMT-NS_ groups did not differ after fecal microbiota transplantation, indicating that pancreatic damage after STZ injection was attributed to the presence of gut microbiota rather than their structural variation.

### STZ-induced pancreatic islet apoptosis due to translocation of gut microbiota independent of its structural variation

**Fig 5:**
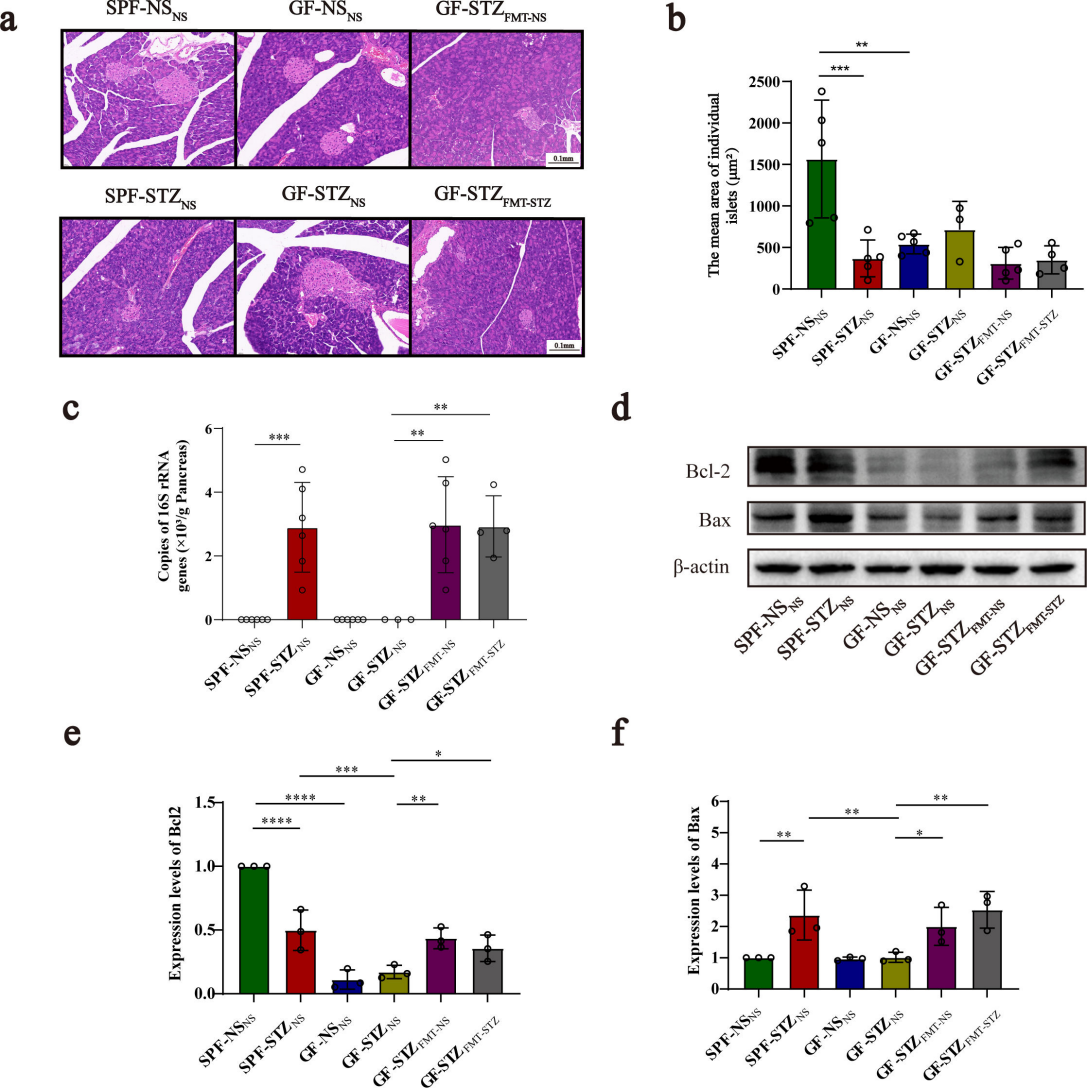
STZ-induced pancreatic islet apoptosis following translocation of gut microbiota independent of its structural variation. (**a**) Pancreas H&E staining. (**b**) The mean area of individual islets. (**c**) The bacterial content in the pancreas was analyzed by qPCR. (**d**) Western blot analysis of pancreatic Bcl-2 and Bax, *n* = 3. (**e**) The relative expression level of Bcl-2 in the pancreas was determined by normalizing Bcl-2 protein levels to β-actin. (**f**) The relative expression level of Bax in the pancreas was determined by normalizing Bax protein levels to β-actin.

Histological examination of the pancreas revealed that GF-STZ_FMT-STZ_ and GF-STZ_FMT-NS_ groups resulted in indistinct visualization of islet boundaries, accompanied by evident damage to islet cells (Fig. 5a). Furthermore, the number of islets and the relative area of islets were significantly lower in GF-STZ_FMT-STZ_ and GF-STZ_FMT-NS_ groups compared with the GF-STZ_NS_ group (Fig. 5b and c).

No discernible disparity was observed in pancreatic bacterial content between the GF-STZ_FMT-STZ_ and GF-STZ_FMT-NS_ groups. No bacteria could be detected in the pancreas in the SPF-STZ_NS_ group (Fig. 5d). These results combined with the suggestion that apoptosis of STZ-induced islet cells depended on gut microbiota translocation.

Furthermore, expressions of anti-apoptotic protein Bcl-2 and apoptotic protein Bax in the pancreas were subsequently assessed using Western blot. The expression of Bcl-2 was significantly lower in the SPF-STZ_NS_ group compared with the SPF-NS_NS_ group (Fig. 5e), while the expression of Bax was higher in the SPF-STZ_NS_ group. The expression levels of Bcl-2 and Bax were comparable between the GF-STZ_NS_ and GF-NS_NS_ groups. These findings were in line with the histological results, suggesting the absence of apoptosis in islets of GF mice following STZ injection. Although there was no significant difference in the expression of apoptosis-related proteins between the GF-STZ_FMT-STZ_ and GF-STZ_FMT-NS_ groups, their Bcl-2 expressions were higher than in the GF-STZ_NS_ group, while Bax expressions elevated significantly compared with that in the GF-STZ_NS_ group. These findings demonstrate that the apoptosis of islet β cells induced by STZ depends on the presence, but not the structure, of the gut microbiota.

### STZ-induced pancreatic damage is accompanied by impairment of intestinal barrier function

**Fig 6:**
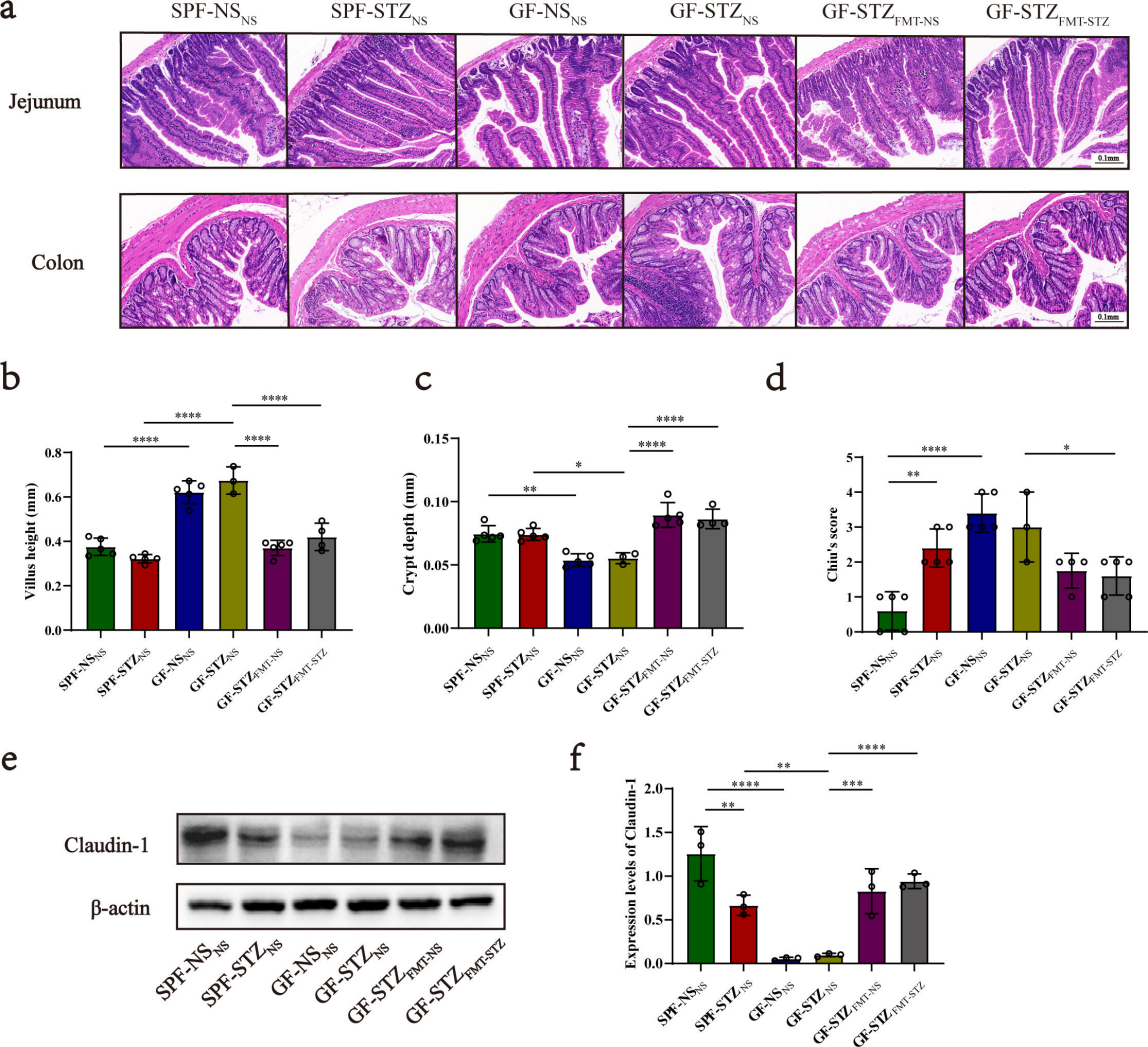
STZ-induced pancreatic damage was accompanied by impairment of intestinal barrier function. (**a**) H&E staining of the jejunum and colon of mice in each group. (**b**) The height of the villi in the jejunum. (**c**) The depth of the crypt in the jejunum. (**d**) Chiu’s score of the small intestine. (**e**) Western blot analysis of tight junction protein Claudin-1 in jejunum, *n* = 3. (**f**) The relative expression level of Claudin-1 in the jejunum was determined by normalizing Claudin-1 protein levels to β-actin.

To investigate the impact of STZ-induced gut microbiota on intestinal barrier function and elucidate the interplay among gut microbiota structure, function, intestinal barrier integrity, and translocation of the microbiota, we evaluated the integrity of intestinal tissues in mice (Fig. 6a). The structure of the jejunum showed improvements following FMT in both the GF-STZ_FMT-STZ_ and GF-STZ_FMT-NS_ groups compared with the GF-STZ_NS_ group. However, no significant difference could be observed between the two FMT groups (Fig. 6a through c). The Chiu’s score of the small intestine exhibited a significant decrease in the two FMT groups compared with that in the GF-STZ_NS_ group revealing that small intestinal barrier improved after FMT (Fig. 6d). Histological analysis of the colon revealed an improvement in the structural integrity of both the GF-STZ_FMT-STZ_ and GF-STZ_FMT-NS_ groups, with no discernible difference observed between the two groups. These results suggested that alterations in the gut microbiota induced by STZ were not the primary cause of intestinal injury, but rather the concurrent change. Conversely, these alterations in the gut microbiota, as the responder to the impairment of the intestinal barrier, may confer protective effects.

The expression of Claudin-1 in the jejunum was assessed using Western blot. The results demonstrated a significant decrease in the expression of Claudin-1 in the SPF-STZ_NS_ group compared with the SPF-NS_NS_ group, and an even lower level in GF mice (GF-STZ_NS_, GF-NS_NS_) (Fig. 6e). Claudin-1 levels in GF-STZ_FMT-STZ_ and GF-STZ_FMT-NS_ groups were also significantly higher than those in the GF-STZ_NS_ group, while there was no difference between the two FMT groups. These findings indicated that the impairment of intestinal barrier was independent of the alteration of gut microbiota induced by STZ.

The intestinal absorption of sugars was evaluated by utilizing liquid-phase mass spectrometry to quantify sugar levels in fecal samples (Fig. S2, S3a through f). There were no discernible disparities in fecal sugars between the SPF-STZ_NS_ group and SPF-NS_NS_ group. Fecal sugar levels in GF mice were significantly higher than those in SPF mice. There was no significant difference between GF-STZ_FMT-STZ_ and GF-STZ_FMT-NS_ groups, which were consistent with those found in SPF mice. These findings suggested the presence of gastrointestinal malabsorption of sugars in GF mice. Although no damage was observed in the islets of GF mice, failure to promote insulin secretion may be associated with diminished intestinal absorption of sugars.

The expression of a sugar transporter GLUT2 was then examined using western blot (Fig. S3g). Expressions of GLUT2 in two GF groups were significantly higher compared with those in the SPF and FMT groups. The increased expression of the sugar transporter GLUT2 in the small intestine suggests that this may be a feedback to the elevated fecal sugar contents.

## DISCUSSION

It is established that by the time of T2DM diagnosis, pancreatic β-cell function is often reduced to less than 50% of normal capacity (36). Following diagnosis, both β-cell mass and function continue to decline progressively. Previous studies have considered that the decomposition of STZ directly induced necrosis or apoptosis in mouse islet cells (20), subsequently leading to diabetes. In the current study, GF mice treated by STZ did not exhibit the dysbiosis of glucose metabolism, suggesting that gut microbiota plays a vital role in the development of STZ-induced diabetes. On the one hand, following STZ injection, GF mice did not exerted intestinal and islet damage or glucose metabolism disorder. Conversely, the results from conventional mice and mice receiving microbiota transplantation demonstrated that in the presence of gut microbiota, STZ induced detrimental effects on the intestinal barrier, microbiota translocation, islet function impairment, and finally led to the development of diabetes. Therefore, our findings validated that STZ indirectly triggers islet apoptosis by impairing the integrity of the intestinal barrier and promoting translocation of intestinal microbiota. On the other hand, despite STZ-induced alterations in the microbiota, no discernible differences were observed in intestinal barrier function, microbiota translocation, islet damage, and glucose metabolism disorder between STZ-injected mice receiving fecal transplantation from either STZ-treated or NS-treated mice. These findings suggested that translocation of microbiota occurs in the presence of intestinal barrier damage, and the extent of intestinal barrier and islet damage caused by this translocation remains unaffected by STZ-induced alterations in intestinal microbiota composition.

We initially hypothesized that the normoglycemia observed in GF mice could be attributed to intact islets and enhanced insulin secretion. Surprisingly, our results demonstrated a notable reduction in insulin secretion in GF mice whether following STZ injection or not. It has been postulated that dietary restriction of sugars not only contributes to the stabilization or reduction of blood glucose levels but also facilitates a decrease in circulating insulin (37). The increased sugar content in the feces of germ-free mice indicated that the gut microbiota played a crucial role in facilitating sugar absorption, which subsequently promoted insulin secretion. This finding aligned with the established understanding that nutrient absorption could stimulate insulin release. However, a critical aspect overlooked by previous studies were that in the absence of gut microbiota—such as in germ-free mice—the toxic effects of STZ were markedly diminished or even abolished, leading to minimal islet damage despite reduced insulin secretion. This phenomenon may be attributed to decreased nutrient absorption. Thus, “reduced sugar absorption” and “impaired STZ efficacy” are not independent outcomes but rather interconnected consequences stemming from the shared underlying factor: the absence of gut microbiota. Our data lead us to a key interpretation that integrates both this observation and the STZ response: the absence of a gut microbiota creates a physiological state characterized by markedly reduced nutrient absorption. This state has two critical, interdependent consequences: (i) It directly leads to diminished insulin secretion due to lack of nutrient stimulus; (ii) More importantly, it prevents the uptake of STZ into pancreatic beta-cells, thereby abolishing its cytotoxic effect. Therefore, “reduced sugar absorption” and “abolished STZ efficacy” are not separate outcomes but are interconnected phenomena both stemming from the fundamental absence of intestinal microbiota. This explains why germ-free mice are protected from diabetes in our model—not because STZ fails to act on its cellular target but because it never reaches the target in sufficient quantities.

Our findings confirmed that impaired sugar absorption resulting from the injury of intestinal barrier was the cause of reduced insulin secretion in GF mice. Additionally, an upregulation of the sugar transporter GLUT2 was observed in the small intestine, suggesting a potential negative feedback mechanism.

It has been reported that STZ may perturb the gut microbiota homeostasis and enhance the abundance of *Oscillospira,* which may compromise the integrity of the intestinal barrier, subsequently leading to microbiota translocation (21). However, we found that short-term injection of STZ could increase the abundance of *A. muciniphila*, which had been reported to maintain intestinal barrier homeostasis (38, 39). Additionally, the content of lysophosphatidic acid that could repair the intestinal barrier (40, 41) also increased after STZ injection. Contrary to previous findings indicating a reduced abundance of *A. muciniphila* long after STZ treatment, our study revealed an augmented presence of *A. muciniphila* shortly after STZ treatment, concomitant with intestinal barrier damage. However, another study had demonstrated consistent findings, indicating an elevation in the abundance of the beneficial bacterium *A. muciniphila* during acute alterations in gastrointestinal functionality (42). We speculated these inconsisitent results may be attributed to the utilization of abundant mucin by *A. muciniphila* derived from relatively mild damage to the intesinal barrier shortly after STZ treatment. Therefore, the increase of *A. muciniphila* and lysophosphatidic acid may represent a concomitant change, potentially indicative of a negative feedback mechanism.

Previous studies have also examined long-term microbial changes after STZ administration (43). These studies mainly discuss the relationship between the changes in the microbiota after a period of STZ injection and the progression of diabetes. However, how early-stage diabetic alterations in the gut microbiota mechanistically contribute to disease onset remains to be elucidated. Our study innovatively captured the acute alterations in microbiota composition during the early phase following STZ injection. By delineating the initial microbial shifts that may contribute to or signal subsequent metabolic decline, these findings provide novel insights into the temporal dynamics of diabetes pathogenesis.

Various environmental endocrine disruptors, such as polychlorinated biphenyls (44, 45), arsenic (46, 47), and cadmium (48, 49), have been found to impair gut function and compromise islet β cells, thereby contributing to the pathogenesis of diabetes. It is still unknown whether such a mechanism is universal in the development of diabetes. In our study, we observed an association between STZ-induced diabetes and both intestinal barrier and islet injuries, which resulted in gut microbiota translocation. These findings had significant implications for understanding the potential role of compounds in impairing intestinal barrier integrity, promoting microbiota migration, damaging pancreatic islets, and finally contributing to the development of diabetes.

The gut microbiota–intestinal barrier–β-cell apoptosis axis was crucial in diabetes pathogenesis. Our study demonstrated that STZ-induced pancreatic damage was linked to gut microenvironment disruption. Unlike previous studies highlighting correlations between gut dysbiosis and the progression of diabetes (50), our study investigated its short-term impact, thereby elucidating the potential involvement of gut microbiota in the early stages of diabetes onset. Whereas existing research emphasized the direct influence of gut microbiota composition on diabetes pathogenesis, this study redirected the focus to the role of microbiota translocation in the initiation and development of diabetes. Importantly, microbiota translocation occurred independently of overall changes in gut microbial composition and was directly initiated by intestinal barrier dysfunction, indicating a distinct pathological pathway that operated separately from structural alterations in the microbiota. These findings provided novel insights into the temporal dynamics of diabetes pathogenesis. This mechanism clarifies how gut-derived factors contribute to diabetes progression in chemical-induced models. Our findings suggest that monitoring markers of gut barrier integrity and bacterial translocation, rather than just microbial composition, could hold promise as a predictive strategy for identifying individuals at high risk for rapid beta-cell loss. This could open avenues for future research into early intervention strategies aimed at preserving intestinal barrier function to delay or prevent diabetes onset. Emerging human data support this perspective. A study of new-onset T1D children showed gut microbiota alterations, including reduced butyrate production and increased virulence factors (13). Many other studies suggested that short-chain fatty acids and virulence factors played a key role in diabetes; however, our findings indicated that gut microbiota may be the primary contributing factor. Although using an STZ model, our results align with human T1D findings, suggesting gut barrier dysfunction may be a common feature across diabetes types—offering a unified target for future research and therapy.

Given that existing literature indicates that a high-fat diet can alter the composition of the gut microbiota, the experimental group received STZ injections in addition to the high-fat diet, with the aim of investigating how STZ-induced microbial changes influence the development of diabetes. However, it cannot be excluded that an interaction exists between the high-fat diet and STZ regarding their combined effects on microbiota composition. In addition, the expression of islet apoptosis-related proteins examined in this study may be influenced by alterations in their upstream regulatory factors. Islet cells integrate various signals from within (such as DNA damage) and outside (such as growth factor signals), change the quantities of BCL-2 and BAX through transcription factors, and directly regulate their activities through BH3-only proteins, ultimately forming a precise balance between BCL-2 and BAX to determine whether the cells survive or undergo apoptosis.

Finally, while we cannot entirely rule out some cage effect in this study, the robust and treatment-associated differences we observed (e.g., in microbiota translocation) are likely driven predominantly by the STZ intervention, given our careful randomization procedure, and we also think that the presence of viable bacteria through culture would provide the most direct evidence. Therefore, in future work, the viability of translocated bacteria in pancreatic islets will be determined using culture-based methods.

In conclusion, the apoptosis of pancreatic β cells in STZ-induced diabetic mice is a consequence of gut microbiota translocation to the pancreas through impaired intestinal barrier, independent of alterations in the gut microbiota.

## Supplementary Material

Reviewer comments

## Data Availability

The metagenomic sequencing data generated in this study have been deposited in the NCBI Sequence Read Archive (SRA) under BioProject accession number PRJNA1467345. The SRA run ID is SRP701447. Data are publicly available as of the date of publication.

## SUPPLEMENTAL MATERIAL

Supplemental Figures msystems.00172-26-s0001.doc Figures S1-S3.

Captions msystems.00172-26-s0002.doc Descriptions of Tables S1-S4.

Supplemental Tables msystems.00172-26-s0003.xlsx Tables S1-S4.

OPEN PEER REVIEW reviewer-comments.pdf An accounting of the reviewer comments and feedback.

ASM does not own the copyrights to Supplemental Material that may be linked to, or accessed through, an article. The authors have granted ASM a non-exclusive, world-wide license to publish the Supplemental Material files. Please contact the corresponding author directly for reuse.
