# Supplementary material for: Gut microbiota translocation contributes to early islet apoptosis in streptozotocin-induced diabetes
Source: mSystems. 2026 Jun 22;11(7):e00172-26. doi: 10.1128/msystems.00172-26 (PMC13386905; doi:10.1128/msystems.00172-26)
Supplement: Supplemental Figures — Figures S1-S3. [file msystems.00172-26-s0001.doc]

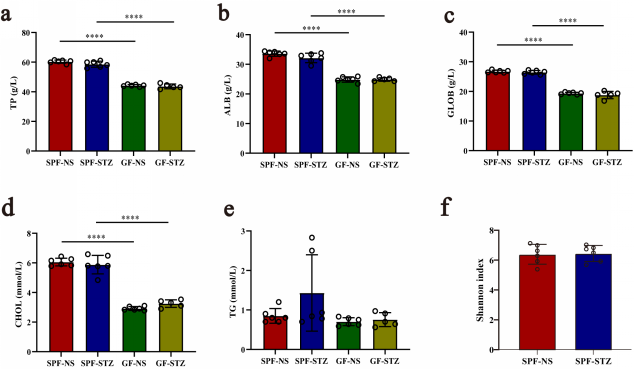
Figure S1 The contents of a) total protein, b) albumin and c) globulin in serum. d) Serum cholesterol and e) triglyceride level, n ≥ 5. f) Shannon index in feces of SPF-NS and SPF-STZ mice.


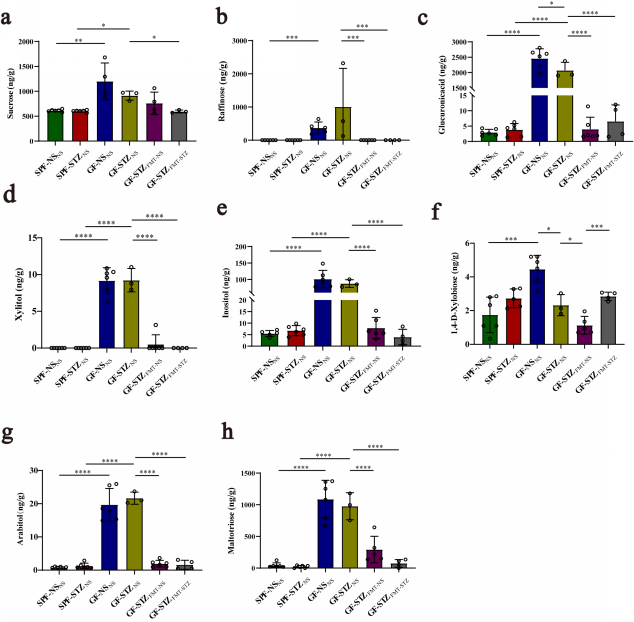


Figure S2 The composition of monosaccharides and oligosaccharides in fecal samples varied among different groups of mice. Levels of a) Sucrose. b) Raffinose. c) Glucuronicacid. d) Xylitol. e) Inositol. f) 1,4-D-xylobiose. g) Arabitol. h) Maltotriose. levels in fecal samples, n ≥ 3.


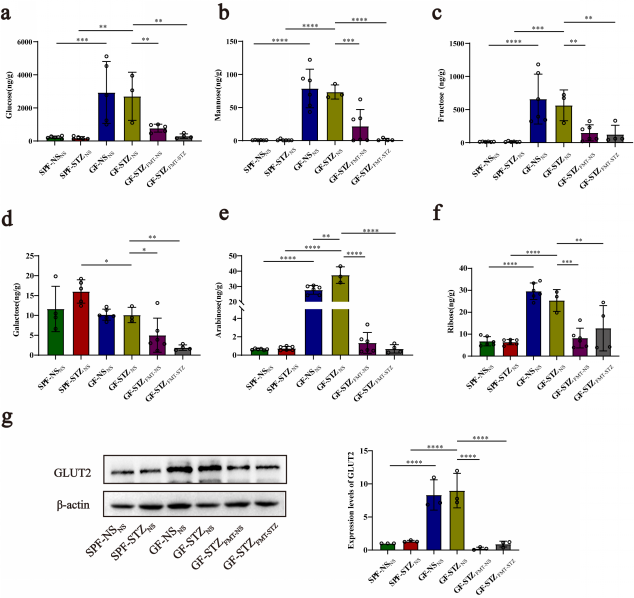
Figure S3 The gut microbiota facilitated the absorption of sugars independent of STZ-induced alterations of the gut microbiota. Levels of a) Glucose. b) Mannose. c) Fructose. d) Galactose. e) Arabinose. f) Ribose levels in fecal samples. g) Western Blot analysis of sugar transporter GLUT2 in jejunum, n ≥ 3.
