# Supplementary material for: Gut microbiota translocation contributes to early islet apoptosis in streptozotocin-induced diabetes
Source: mSystems. 2026 Jun 22;11(7):e00172-26. doi: 10.1128/msystems.00172-26 (PMC13386905; doi:10.1128/msystems.00172-26)
Supplement: Captions — Descriptions of Tables S1-S4. [file msystems.00172-26-s0002.doc]

S1: The raw data for the metagenomic results in Figure 3a, e, h.

S2: The raw data for the metagenomic results in Figure 3c (LDA score＞2).

S3: The raw data for the metagenomic results in Figure 3f (LDA score＞2).

S4: The raw data for the metagenomic results in Figure 3i (LDA score＞2).
