# Supplementary material for: Gut microbiota translocation contributes to early islet apoptosis in streptozotocin-induced diabetes
Source: mSystems. 2026 Jun 22;11(7):e00172-26. doi: 10.1128/msystems.00172-26 (PMC13386905; doi:10.1128/msystems.00172-26)
Supplement: Reviewer comments [file reviewer-comments.pdf]

AMERICAN SOCIETY FOR MICROBIOLOGY | mSystems®

Gut Microbiota Translocation Contributes to Early Islet Apoptosis iStreptozotocin-Induced Diabetes

Feng Zhang, Ying Wang, Peng Sheng, Shijia Wang, Xiaohui Zhong, Hong Cao, Dan Li, Jiai Yan, Ju Yang, Yingyu Wang, Jie Peng, Jing Sun, Shunhe Wang, Fengping Sun, and Yongwei Feng

Corresponding Author(s): Feng Zhang, Affiliated Hospital of Jiangnan University

Review Timeline:

Submission Date:	February 22, 2026
Accepted:	April 22, 2026

Editor: Sophie Darch

Reviewer(s): Disclosure of reviewer identity is with reference to reviewer comments included in decision letter(s). The following individuals involved in review of your submission have agreed to reveal their identity: Elliot S Friedman (Reviewer #3)

Transaction Report:

(Note: With the exception of the correction of typographical or spelling errors that could be a source of ambiguity, letters and reports are not edited. The original formatting of letters and referee reports may not be reflected in this compilation.)

DOI: https://doi.org/10.1128/msystems.00172-26

Re: mSystems00172-26 (**Gut Microbiota Translocation Contributes to Early Islet Apoptosis in Streptozotocin-Induced Diabetes**)

Dear Dr. Feng Zhang:

Your manuscript has been accepted, and I am forwarding it to the ASM production staff for publication. Your paper will first be checked to make sure all elements meet the technical requirements. ASM staff will contact you if anything needs to be revised before copyediting and production can begin. Otherwise, you will be notified when your proofs are ready to be viewed.

Data Availability: ASM policy requires that data be available to the public upon online posting of the article, so please verify all links to sequence records, if present, and make sure that each number retrieves the full record of the data. If a new accession number is not linked or a link is broken, provide production staff with the correct URL for the record. If the accession numbers for new data are not publicly accessible before the expected online posting of the article, publication may be delayed; please contact ASM production staff immediately with the expected release date.

Publication Fees: For information on publication fees and which article types have charges, please visit our website. We have partnered with Copyright Clearance Center (CCC) to collect author charges. If fees apply to your paper, you will receive a message from no-reply@copyright.com with further instructions. For questions related to paying charges through RightsLink, please contact CCC at ASM_Support@copyright.com or toll free at +1-877-622-5543. CCC makes every attempt to respond to all emails within 24 hours.

ASM Membership: Corresponding authors may join or renew ASM membership to obtain discounts on publication fees. Need to upgrade your membership level? Please contact Customer Service at Service@asmusa.org.

Embargo Policy: A press release may be issued as soon as the manuscript is posted on the mSystems Latest Articles webpage. The corresponding author will receive an email with the subject line "ASM Journals Author Services Notification" when the article is available online.

Cover Image Submissions: If you would like to submit a potential Cover Image, please email a file and a short legend to msystems@asmusa.org. Please note that we can only consider images that (i) the authors created or own and (ii) have not been previously published. By submitting, you agree that the image can be used under the same terms as the published article. Image File requirements: TIF/EPS, 7.5 inches wide by 8.25 inches tall (at least 2,250 pixels wide by 2,475 pixels tall), minimum 300 dpi resolution (600 dpi preferred), RGB, and no figure elements, e.g., arrows or panel labels. The legend should be a short description of the image, 1-2 sentences recommended. Please download and use this interactive template in Adobe to ensure that your proposed cover image meets our size requirements (https://journals.asm.org/pb-assets/pdf-text-excel-files/ASM-Interactive-Sizing-Cover-Template-1715689791.pdf).

Thank you for submitting your paper to mSystems.

Sincerely,
Sophie Darch
Editor
mSystems

Reviewer #1 (Comments for the Author):

good work

Reviewer #3 (Comments for the Author):

I appreciate the work that went into repeating the experiment to increase the sample size. I see that this is noted in Fig 1 and 2.

Figure 4 - what does "n greater than or equal to 3" mean?
Figure 5 and 6 - Please note sample size in figure legend.

Also the supplemental figures.
